# First crystal structure of a Pigment Red 52 compound: DMSO solvate hydrate of the monosodium salt

**DOI:** 10.1107/S2056989021002577

**Published:** 2021-03-19

**Authors:** Lukas Tapmeyer, Daniel Eisenbeil, Michael Bolte, Martin U. Schmidt

**Affiliations:** a Institut für Anorganische und Analytische Chemie, Goethe-Universität Frankfurt, Max-von-Laue-Strasse 7, 60438 Frankfurt am Main, Germany

**Keywords:** crystal structure determination, organic pigment, solvate, hydrate

## Abstract

Pigment Red 52, Na_2_[C_18_H_11_ClN_2_O_6_S], is an industrially produced hydrazone-laked pigment. It serves as an inter­mediate in the synthesis of the corresponding Ca^2+^ and Mn^2+^ salts, which are used commercially for printing inks and lacquers. Hitherto, no crystal structure of any salt of Pigment Red 52 is known. Now, single crystals have been obtained of a dimethyl sulfoxide solvate hydrate of the monosodium salt of Pigment Red 52, namely, monosodium 2-[2-(3-carb­oxy-2-oxo-1,2-di­hydro­naphthalen-1-yl­idene)hydrazin-1-yl]-5-chloro-4-methyl­benz­ene­sulfonate dimethyl sulfoxide monosolvate monohydrate, Na^+^·C_18_H_12_ClN_2_O_6_S^−^·H_2_O·C_2_H_6_OS, obtained from in-house synthesized Pigment Red 52. The crystal structure was determined by single-crystal X-ray diffraction at 173 K. In this monosodium salt, the SO_3_^−^ group is deprotonated, whereas the COOH group is protonated. The residues form chains *via* ionic inter­actions and hydrogen bonds. The chains are arranged in polar/non-polar double layers.

## Abstract 2

The crystal structure of a DMSO monosolvate monohydrate of a previously unknown monosodium salt of the industrial inter­mediate Pigment Red 52 (P.R.52) with the formula Na^+^[C_18_H_12_ClN_2_O_6_S]^−^·H_2_O·C_2_H_6_OS. The compound was obtained by in-house synthesis. The crystals have triclinic symmetry at 173 K. The crystal structure is built up by Na—O chains, which arrange the anions in polar/non-polar double layers.

## Chemical context   

Pigment Red 52 (P.R.52, Na_2_[C_18_H_11_N_2_ClO_6_S]), is produced industrially as an inter­mediate in the synthesis of Pigment Red 52:1 (Ca[C_18_H_11_N_2_ClO_6_S]) and Pigment Red 52:2 (Mn[C_18_H_11_N_2_ClO_6_S]) (Czajkowski *et al.*, 1980 ▸; Hunger & Schmidt, 2018 ▸). P.R.52:1 and P.R.52:2 are used for the colouration of printing inks and lacquers (Hunger & Schmidt, 2018 ▸). No crystal structures of P.R.52, or of its various metal salts, have previously been determined. Pigment Red 48 is an isomer of P.R.52, differing by mutual exchange of CH_3_ and Cl substituents. Recently, the crystal structures of two hydrates of the monosodium salt of P.R.48 have been published (Tapmeyer *et al.*, 2020 ▸). Correspondingly, similar monosodium hydrate phases could also be expected for P.R.52. Hitherto, nothing has been known about the existence of a monosodium salt of P.R.52 or its hydrates or solvates. In attempts to crystallize P.R.52 from di­methyl­sulfoxide, single crystals were obtained, which turned out to be a mono-DMSO solvate monohydrate of the monosodium salt of P.R.52:1. The crystal structure was determined by X-ray analysis.

## Structural commentary   

**Figure 1:**
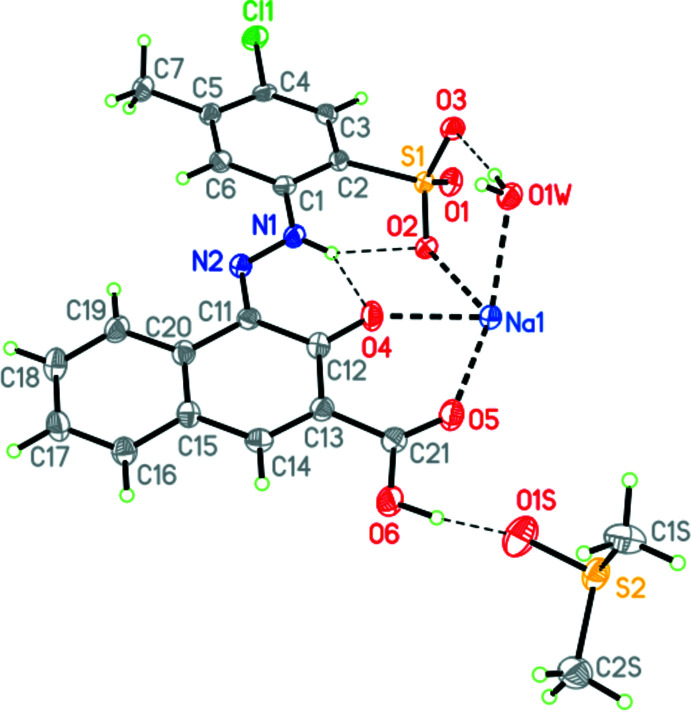
A perspective view of the asymmetric unit of the title compound. Displacement ellipsoids are drawn at the 50% probability level.

Pigment Red 52 monosodium salt DMSO monosolvate monohydrate crystallizes in the triclinic space group *P* 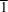 with one pigment anion, one sodium cation, one mol­ecule of DMSO and one water mol­ecule in the asymmetric unit (Fig. 1 ▸).
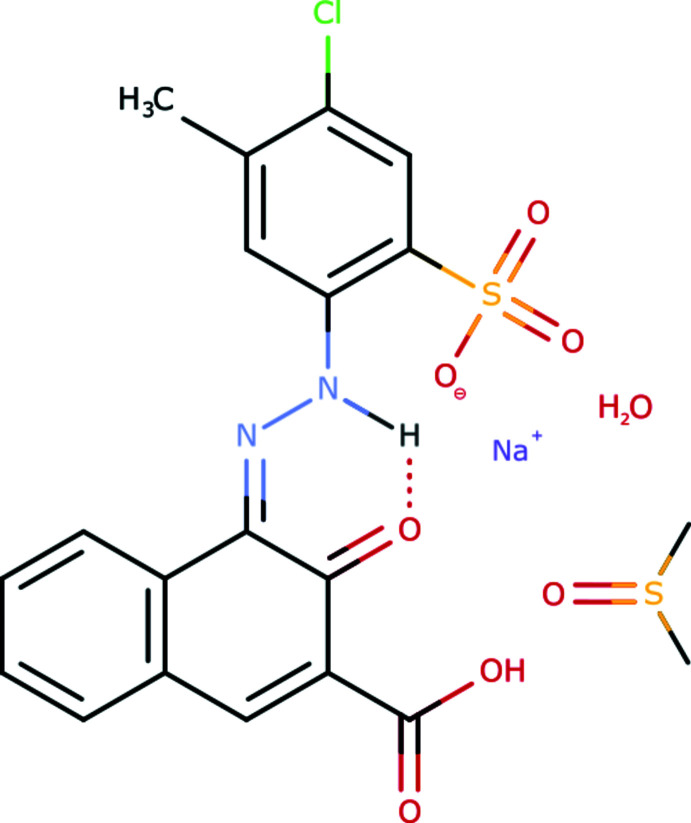


The pigment exhibits the hydrazone tautomeric form, like all industrial hydrazone pigments (formerly known as ‘azo pigments’) (Gilli *et al.*, 2005 ▸; Schmidt *et al.*, 2008 ▸; Hunger & Schmidt, 2018 ▸). The N—H group forms two intra­molecular [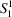
(6)] N—H⋯O hydrogen bonds (Table 1 ▸). The sulfonate group is deprotonated, whereas the carb­oxy­lic group is protonated. The protonation site is unambiguously determined by the difference electron density, from the S—O and C—O bond lengths in the SO_3_^−^ and COOH groups, and from the hydrogen-bond pattern. Intra­molecular and inter­molecular bond lengths and angles are in the usual ranges. The organic anion is nearly planar, with an RMSD of 0.553 Å for all non-hydrogen atoms, except for the oxygen atoms of the sulfonate group. The dihedral angle between the naphthyl moiety and the phenyl ring is 9.84 (16)°.

The carb­oxy­lic acid group is coplanar with the naphthyl moiety [dihedral angle of 1.2 (5)°, see Fig. 1 ▸]. This coplanarity is a peculiarity, as in most other related structures, the COOH group is rotated out of the naphthyl plane (Table 2 ▸).

## Supra­molecular features   

**Figure 2:**
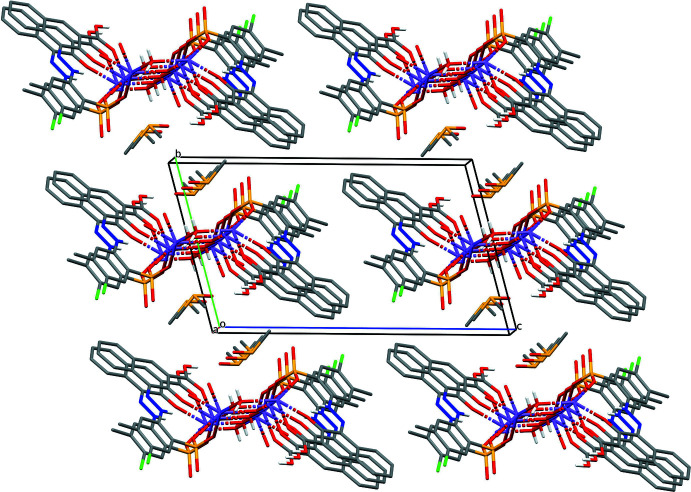
Packing diagram viewed approximately along [100].

**Figure 3:**
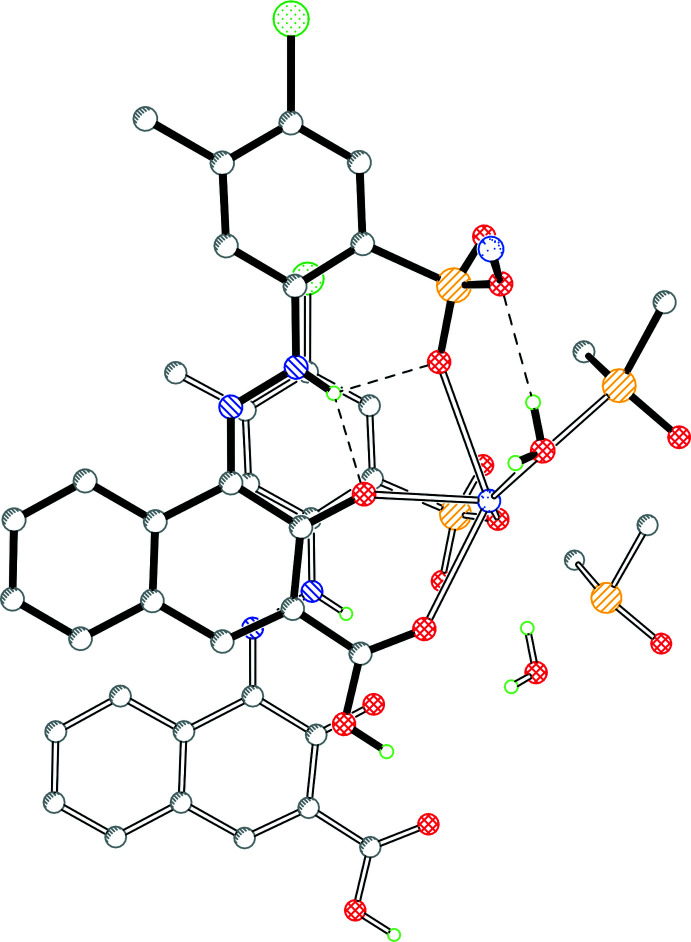
π-stacking of two anions, one drawn with full bonds and the other one with open bonds.

The protonated carboxyl oxygen atom of the COOH group donates a hydrogen bond to the DMSO mol­ecule (Table 1 ▸). The other carboxyl oxygen atom accepts a hydrogen bond from the water mol­ecule and additionally coordinates to the sodium ion. The sodium ion is sixfold coordinated to one oxygen atom of the COOH group, the carbonyl group, an oxygen atom of the sulfonate group, and two water mol­ecules (one belonging to the same asymmetric unit, the other one transformed by −*x*, 1 − *y*, −*z*). The sixth coordination site is occupied by an O atom of a sulfonate group of a neighbouring anion, generated by the symmetry operation −1 + *x*, *y*, *z*. The coordination polyhedron is a distorted octa­hedron. The crystal packing is characterized by chains built *via* Na—O coordinations, running along the *a-*axis direction (Fig. 2 ▸). Within this chain, the phenyl ring is π-stacked above the O=C—C=N—N—H moiety of a symmetry-equivalent anion (1 + *x*, *y*, *z*) with the shortest distance C6⋯C12 of 3.303 (5) Å. The N–NH unit is stacked above the naphthyl-COOH group with the shortest distance N1⋯C21 (1 + *x*, *y*, *z*) of 3.304 (4) Å (Fig. 3 ▸).

## Database survey   

For Pigment Red 52 and its derivatives, this is the first crystal structure published. Some closely related structures are compared in Table 2 ▸, *viz*. bis­[6-chloro-3-(3-carb­oxy-2-oxo­anthracenylidenehydrazono)benzene­sulfonato]­bis­(di­methyl­formamide)­calcium (BIHNUC; Kennedy *et al.*, 2004 ▸), [4-(4,6-di­chloro-2-sulfophen­yl)azo-3-hy­droxy-2-naphtho­ato]di­aqua­calcium (KAQSAW; Kennedy *et al.*, 2000 ▸), {3-carb­oxy-1-[2-(5-chloro-4-methyl-2-sulfophen­yl)diazen-2-ium-1-yl]naphthalen-2-olato}di­aqua­sodium, {3-carb­oxy-1-[2-(5-chloro-4-methyl-2-sulfophen­yl)diazen-2-ium-1-yl]naphthalen-2-olato}-aqua-sodium (GUNZAT and GUNZEX, respectively; Tapmeyer *et al.*, 2020 ▸), {3-carb­oxy-1-[2-(4-methyl-2-sulfophen­yl)diazen-2-ium-1-yl]naphthalen-2-olato}calcium, {2-[2-(3-carb­oxy-2-oxy-1-naphth­yl)diazenium­yl]-5-methyl­benzene­sulfonato}­tri­aqua­calcium, {2-[2-(3-carb­oxy-2-hy­droxy-1-naphth­yl)diazenium­yl]-5-methyl­benzene­sulfonato}­aqua­calcium (FAWQUR, FAWQIF and FAWQOL, respectively; Bekö *et al.*, 2012*a* ▸,*b* ▸), bis­(3-oxido-4-[(1*H*-1,2,4-triazol-3-yl)diazen­yl]naphthalene-2-carboxyl­ato)bis­(3-hy­droxy­naphthalene-2-carboxyl­ato)tetra­kis­(aqua)­didysprosium(III) *N*,*N*-di­methyl­formamide solvate, bis­{3-oxido-4-[(1*H*-1,2,4-triazol-3-yl)diazen­yl]naphthalene-2-carboxyl­ato}bis­(3-hy­droxy­naphthalene-2-carboxyl­ato)tetra­kis­(aqua)­dieuropium(III) *N*,*N-*di­methyl­formamide solvate, bis­{3-oxido-4-[(1*H*-1,2,4-triazol-3-yl)diazen­yl]naphthalene-2-carboxyl­ato}bis­(3-hy­droxy­naphthalene-2-carboxyl­ato)tetra­kis­(aqua)­diterbium(III) *N*,*N*-di­methyl­formamide solvate, bis­(3-oxido-4-[(1*H*-1,2,4-triazol-3-yl)diazen­yl]naphthalene-2-carboxyl­ato)bis­(3-hy­droxy­naphthalene-2-carboxyl­ato)tetra­kis­(aqua)­disamarium(III) *N*,*N*-di­methyl­formamide solvate (BOGDUZ, BOGFIP, BOGFAH and BOGFEL, respectively; Xie *et al.*, 2019 ▸).

## Synthesis and crystallization   

The title compound was obtained by recrystallization experiments of in-house synthesized P.R.52.

### Synthesis of Pigment Red 52   

2-Amino-5-chloro-*p*-toluene­sulfonic acid (22.15 g, 0.1 mol) was dissolved with sodium hydroxide (6.4 g) in water (500 ml). The temperature was set at 278 K and concentrated hydro­chloric acid (40 ml) as well as sodium nitrite (7.2 g) in water (100 ml) were added. The suspension was stirred for 30 min. The suspension was treated with amido­sulfonic acid until all excess nitrous acid was destroyed. The suspension was then added dropwise to a solution of β-oxynaphthoic acid (18.8 g, 0.1 mol) with NaOH (20.1 g) in water (550 ml). The pH was kept at alkaline conditions, around 11 to 9, maintained by the addition of 2 *M* NaOH solution as required, and the temperature was maintained at 278 K. When the dropwise addition of the suspension was finished, the solution was allowed to accommodate to room temperature and subsequently heated to 353 K for half an hour. The red suspension was then neutralized with 2 *M* HCl, filtered off and the obtained red powder was washed with water and dried at 323 K. The yield of the crude product was about 98%, but X-ray powder diffraction revealed the presence of some sodium chloride as impurity.

### Crystallization of the title compound   

The crude in-house synthesized P.R.52 (0.59 g) was dissolved in DMSO (60 ml). The solution was transferred to a glass vessel, which in turn was placed into a further, larger vessel with water (100 ml). The outer vessel was closed with a plastic lid and stored for 20 days at room temperature, allowing the water to diffuse into the DMSO *via* the gas phase. Single crystals of the title compound were picked from the solution.

## Refinement   

Crystal data, data collection and structure refinement details are summarized in Table 3 ▸. The H atoms bonded to C were refined using a riding model with C—H = 0.95 Å and with *U*_iso_(H) = 1.2*U*_eq_(C) or with C_meth­yl_—H = 0.98 Å and with *U*_iso_(H) = 1.5*U*_eq_(C). The methyl group attached to the phenyl ring was allowed to rotate but not to tip. The H atoms bonded to N and O were found in the difference-Fourier synthesis and freely refined.

## Supplementary Material

Crystal structure: contains datablock(s) I. DOI: 10.1107/S2056989021002577/yk2145sup1.cif

Structure factors: contains datablock(s) I. DOI: 10.1107/S2056989021002577/yk2145Isup2.hkl

CCDC reference: 2068733

Additional supporting information:  crystallographic information; 3D view; checkCIF report

## Figures and Tables

**Table 1:** Hydrogen-bond geometry (Å, °)

*D*—H⋯*A*	*D*—H	H⋯*A*	*D*⋯*A*	*D*—H⋯*A*
N1—H1⋯O2	0.82 (4)	2.14 (4)	2.747 (3)	131 (3)
N1—H1⋯O4	0.82 (4)	1.84 (4)	2.532 (4)	141 (4)
O6—H6⋯O1*S*	0.92 (5)	1.67 (5)	2.575 (4)	168 (4)
O1*W*—H1*WA*⋯O5^i^	0.80 (5)	2.33 (5)	2.944 (3)	134 (4)
O1*W*—H1*WA*⋯O1*S* ^i^	0.80 (5)	2.48 (5)	3.138 (5)	140 (4)
O1*W*—H1*WB*⋯O3	0.84 (6)	2.11 (6)	2.942 (3)	176 (5)

Symmetry code: (i) x+1, y, z.

**Table 2:** Angles (°) of the C–COO(H) plane to the mean plane of the carbon skeleton of the β-oxynaphthoic acid moiety

Refcode	Salt	Solvate / Hydrate	|Angle|
BIHNUC*^*a*^*	Ca[C_17_H_10_N_2_O_6_ClS]_2_	2 DMF	0.09
GUNZAT	Na[C_18_H_12_ClN_2_O_6_S]	2 H_2_O	2.43
FAWQUR	Ca[C_18_H_12_N_2_O_6_S]		22.5
KAQSAW	Ca[C_17_H_8_C_l2_N_2_O_6_S]	2 H_2_O	23.3
FAWQIF	Ca[C_18_H_14_N_2_O_7_S]	2 H_2_O	26.3
GUNZEX	Na[C_18_H_12_ClN_2_O_6_S]	1 H_2_O	28.6
BOGDUZ*^*a*^*	Dy[C_13_H_7_N_5_O_3_][BONA]*^*b*^*	2 DMF, 2 H_2_O	36.6
FAWQOL	Ca[C_18_H_12_N_2_O_6_S]	1 H_2_O	37.7
BOGFIP*^*a*^*	Eu[C_13_H_7_N_5_O_3_][BONA]*^*b*^*	DMF, 4 H_2_O	39.0
BOGFAH*^*a*^*	Tb[C_13_H_7_N_5_O_3_][BONA]*^*b*^*	DMF, 4 H_2_O	39.0
BOGFEL*^*a*^*	Sm[C_13_H_7_N_5_O_3_][BONA]*^*b*^*	DMF, 4 H_2_O	39.1

Notes: (*a*) This is not a pigment with a Colour Index number; (*b*) BONA = β-oxynaphthoic acid.

**Table 3:** Experimental details

Crystal data	
Chemical formula	Na^+^·C_18_H_12_ClN_2_O_6_S^−^·C_2_H_6_OS·H_2_O
*M*_r_	538.94
Crystal system, space group	Triclinic, *P*\overline{1}
Temperature (K)	173
*a*, *b*, *c* (Å)	5.7347 (4), 10.9336 (8), 18.4692 (12)
α, β, γ (°)	104.844 (5), 97.478 (5), 95.404 (6)
*V* (Å^3^)	1100.00 (14)
*Z*	2
Radiation type	Mo *K*α
μ (mm^−1^)	0.44
Crystal size (mm)	0.23 × 0.09 × 0.02

Data collection	
Diffractometer	STOE IPDS II two-circle
Absorption correction	Multi-scan (*X-AREA*; Stoe & Cie, 2001 ▸)
*T*_min_, *T*_max_	0.445, 1.000
No. of measured, independent and observed [*I* > 2σ(*I*)] reflections	14981, 3864, 2992
*R*_int_	0.049
(sin θ/λ)_max_ (Å^−1^)	0.595

Refinement	
*R*[*F* ^2^ > 2σ(*F* ^2^)], *wR*(*F* ^2^), *S*	0.050, 0.098, 1.08
No. of reflections	3864
No. of parameters	324
H-atom treatment	H atoms treated by a mixture of independent and constrained refinement
Δρ_max_, Δρ_min_ (e Å^−3^)	0.27, −0.33

Computer programs: *X-AREA* (Stoe & Cie, 2001 ▸), *SHELXT* (Sheldrick, 2015*a* ▸), *SHELXL* and *XP* (Sheldrick, 2015*b* ▸), *Mercury* (Macrae *et al.*, 2020 ▸) and *publCIF* (Westrip, 2010 ▸).

## supplementary crystallographic information

### Crystal data

**Table:**

Na^+^·C_18_H_12_ClN_2_O_6_S^−^·C_2_H_6_OS·H_2_O	*Z* = 2
*M**_r_* = 538.94	*F*(000) = 556
Triclinic, *P*1	*D*_x_ = 1.627 Mg m^−^^3^
*a* = 5.7347 (4) Å	Mo *K*α radiation, λ = 0.71073 Å
*b* = 10.9336 (8) Å	Cell parameters from 12767 reflections
*c* = 18.4692 (12) Å	θ = 3.4–27.4°
α = 104.844 (5)°	µ = 0.44 mm^−^^1^
β = 97.478 (5)°	*T* = 173 K
γ = 95.404 (6)°	Needle, red
*V* = 1100.00 (14) Å^3^	0.23 × 0.09 × 0.02 mm

### Data collection

**Table:**

STOE IPDS II two-circle diffractometer	2992 reflections with *I* > 2σ(*I*)
Radiation source: Genix 3D IµS microfocus X-ray source	*R*_int_ = 0.049
ω scans	θ_max_ = 25.0°, θ_min_ = 3.4°
Absorption correction: multi-scan (X-Area; Stoe & Cie, 2001)	*h* = −6→6
*T*_min_ = 0.445, *T*_max_ = 1.000	*k* = −12→12
14981 measured reflections	*l* = −21→21
3864 independent reflections	

### Refinement

**Table:**

Refinement on *F*^2^	0 restraints
Least-squares matrix: full	Hydrogen site location: mixed
*R*[*F*^2^ > 2σ(*F*^2^)] = 0.050	H atoms treated by a mixture of independent and constrained refinement
*wR*(*F*^2^) = 0.098	*w* = 1/[σ^2^(*F*_o_^2^) + (0.0429*P*)^2^ + 0.0815*P*] where *P* = (*F*_o_^2^ + 2*F*_c_^2^)/3
*S* = 1.08	(Δ/σ)_max_ < 0.001
3864 reflections	Δρ_max_ = 0.27 e Å^−^^3^
324 parameters	Δρ_min_ = −0.33 e Å^−^^3^

### Special details

**Table:**

Geometry. All esds (except the esd in the dihedral angle between two l.s. planes) are estimated using the full covariance matrix. The cell esds are taken into account individually in the estimation of esds in distances, angles and torsion angles; correlations between esds in cell parameters are only used when they are defined by crystal symmetry. An approximate (isotropic) treatment of cell esds is used for estimating esds involving l.s. planes.

### Fractional atomic coordinates and isotropic or equivalent isotropic displacement parameters (Å^2^)

**Table:**

	*x*	*y*	*z*	*U*_iso_*/*U*_eq_	
Cl1	1.33825 (14)	0.84822 (8)	0.39943 (5)	0.0267 (2)	
S1	0.52780 (13)	0.72725 (7)	0.20025 (4)	0.01767 (18)	
N1	0.4274 (5)	0.5113 (2)	0.28158 (16)	0.0202 (6)	
H1	0.327 (7)	0.519 (3)	0.248 (2)	0.030 (10)*	
N2	0.3632 (4)	0.4232 (2)	0.31510 (14)	0.0190 (6)	
O1	0.5585 (4)	0.8648 (2)	0.21703 (13)	0.0255 (5)	
O2	0.2857 (4)	0.6713 (2)	0.19634 (12)	0.0233 (5)	
O3	0.6302 (4)	0.6669 (2)	0.13374 (12)	0.0233 (5)	
O4	0.0393 (4)	0.4384 (2)	0.19077 (13)	0.0304 (6)	
O5	−0.3920 (4)	0.3586 (2)	0.10391 (13)	0.0289 (5)	
O6	−0.6211 (4)	0.2111 (2)	0.13512 (14)	0.0318 (6)	
H6	−0.722 (8)	0.211 (4)	0.092 (3)	0.053 (13)*	
C1	0.6441 (5)	0.5895 (3)	0.30879 (17)	0.0182 (6)	
C2	0.7033 (5)	0.6915 (3)	0.27795 (17)	0.0176 (6)	
C3	0.9189 (5)	0.7691 (3)	0.30758 (17)	0.0195 (7)	
H3	0.961076	0.838914	0.287912	0.023*	
C4	1.0722 (5)	0.7460 (3)	0.36520 (18)	0.0192 (7)	
C5	1.0206 (5)	0.6441 (3)	0.39538 (17)	0.0197 (7)	
C6	0.8039 (5)	0.5675 (3)	0.36633 (17)	0.0198 (7)	
H6A	0.763355	0.497883	0.386333	0.024*	
C7	1.1910 (6)	0.6171 (3)	0.45670 (19)	0.0250 (7)	
H7A	1.190008	0.680328	0.505018	0.038*	
H7B	1.351237	0.621942	0.443631	0.038*	
H7C	1.142866	0.531421	0.461406	0.038*	
C11	0.1537 (5)	0.3519 (3)	0.29167 (17)	0.0189 (6)	
C12	−0.0153 (6)	0.3617 (3)	0.22721 (18)	0.0220 (7)	
C13	−0.2443 (5)	0.2803 (3)	0.20857 (18)	0.0200 (7)	
C14	−0.2928 (6)	0.1985 (3)	0.25062 (18)	0.0206 (7)	
H14	−0.441828	0.145890	0.237181	0.025*	
C15	−0.1318 (6)	0.1870 (3)	0.31425 (17)	0.0198 (7)	
C16	−0.1975 (6)	0.1025 (3)	0.35634 (18)	0.0232 (7)	
H16	−0.349077	0.052284	0.342594	0.028*	
C17	−0.0435 (6)	0.0924 (3)	0.41705 (19)	0.0277 (8)	
H17	−0.088209	0.034856	0.445145	0.033*	
C18	0.1790 (6)	0.1664 (3)	0.4379 (2)	0.0289 (8)	
H18	0.284455	0.158951	0.480198	0.035*	
C19	0.2474 (6)	0.2504 (3)	0.39741 (19)	0.0259 (7)	
H19	0.399428	0.300111	0.411918	0.031*	
C20	0.0923 (5)	0.2620 (3)	0.33504 (18)	0.0201 (7)	
C21	−0.4220 (5)	0.2885 (3)	0.14433 (18)	0.0204 (7)	
Na1	−0.0772 (2)	0.53586 (12)	0.09912 (7)	0.0231 (3)	
S2	−1.06094 (15)	0.18210 (8)	−0.06033 (5)	0.0275 (2)	
O1S	−0.8734 (5)	0.1866 (3)	0.00506 (17)	0.0598 (9)	
C1S	−1.0132 (7)	0.0551 (4)	−0.1364 (2)	0.0370 (9)	
H1S1	−1.131988	0.047817	−0.181088	0.055*	
H1S2	−0.854081	0.072376	−0.148510	0.055*	
H1S3	−1.027239	−0.024884	−0.121697	0.055*	
C2S	−1.3286 (7)	0.1091 (4)	−0.0416 (2)	0.0360 (9)	
H2S1	−1.457635	0.103949	−0.083129	0.054*	
H2S2	−1.308418	0.022976	−0.037569	0.054*	
H2S3	−1.367318	0.160165	0.006106	0.054*	
O1W	0.2484 (4)	0.4817 (3)	0.02986 (13)	0.0235 (5)	
H1WA	0.288 (8)	0.415 (5)	0.034 (3)	0.056 (15)*	
H1WB	0.359 (9)	0.536 (5)	0.058 (3)	0.065 (16)*	

### Atomic displacement parameters (Å^2^)

**Table:**

	*U*^11^	*U*^22^	*U*^33^	*U*^12^	*U*^13^	*U*^23^
Cl1	0.0170 (4)	0.0301 (4)	0.0295 (5)	−0.0030 (3)	−0.0035 (3)	0.0075 (3)
S1	0.0161 (4)	0.0210 (4)	0.0164 (4)	0.0031 (3)	0.0008 (3)	0.0066 (3)
N1	0.0148 (13)	0.0246 (14)	0.0211 (15)	−0.0007 (11)	−0.0017 (12)	0.0093 (11)
N2	0.0185 (14)	0.0214 (13)	0.0176 (14)	0.0037 (11)	0.0022 (11)	0.0062 (11)
O1	0.0285 (13)	0.0213 (11)	0.0253 (13)	0.0036 (9)	−0.0013 (10)	0.0067 (9)
O2	0.0157 (11)	0.0332 (12)	0.0229 (12)	0.0025 (9)	0.0006 (9)	0.0123 (10)
O3	0.0200 (11)	0.0314 (12)	0.0184 (12)	0.0077 (9)	0.0034 (9)	0.0052 (9)
O4	0.0238 (12)	0.0412 (14)	0.0280 (13)	−0.0070 (10)	−0.0064 (10)	0.0218 (11)
O5	0.0265 (13)	0.0308 (12)	0.0299 (14)	−0.0025 (10)	−0.0044 (10)	0.0159 (11)
O6	0.0218 (13)	0.0412 (14)	0.0291 (14)	−0.0110 (11)	−0.0098 (11)	0.0162 (11)
C1	0.0155 (15)	0.0198 (15)	0.0182 (16)	0.0020 (12)	0.0033 (12)	0.0029 (12)
C2	0.0182 (15)	0.0204 (15)	0.0151 (16)	0.0044 (12)	0.0030 (12)	0.0058 (12)
C3	0.0182 (16)	0.0214 (16)	0.0183 (16)	0.0019 (13)	0.0039 (13)	0.0042 (12)
C4	0.0111 (15)	0.0246 (16)	0.0196 (17)	0.0013 (12)	0.0021 (12)	0.0024 (13)
C5	0.0189 (16)	0.0232 (16)	0.0170 (17)	0.0061 (13)	0.0027 (13)	0.0043 (13)
C6	0.0177 (16)	0.0221 (15)	0.0186 (17)	0.0001 (12)	0.0006 (13)	0.0055 (13)
C7	0.0213 (17)	0.0307 (17)	0.0221 (18)	0.0036 (14)	−0.0033 (14)	0.0087 (14)
C11	0.0159 (16)	0.0232 (15)	0.0161 (16)	0.0010 (12)	0.0016 (12)	0.0038 (12)
C12	0.0231 (17)	0.0230 (16)	0.0204 (17)	0.0039 (13)	0.0017 (13)	0.0072 (13)
C13	0.0192 (16)	0.0214 (15)	0.0190 (17)	0.0030 (12)	0.0020 (13)	0.0053 (13)
C14	0.0187 (16)	0.0185 (15)	0.0213 (17)	−0.0008 (12)	0.0024 (13)	0.0012 (13)
C15	0.0239 (16)	0.0181 (15)	0.0161 (16)	0.0031 (12)	0.0019 (13)	0.0031 (12)
C16	0.0259 (17)	0.0212 (16)	0.0219 (18)	0.0018 (13)	0.0021 (14)	0.0062 (13)
C17	0.0328 (19)	0.0266 (17)	0.0269 (19)	0.0037 (15)	0.0038 (15)	0.0140 (14)
C18	0.034 (2)	0.0289 (18)	0.0237 (19)	0.0048 (15)	−0.0024 (15)	0.0103 (14)
C19	0.0214 (17)	0.0296 (17)	0.0257 (18)	0.0018 (14)	−0.0024 (14)	0.0095 (14)
C20	0.0222 (16)	0.0198 (15)	0.0188 (17)	0.0057 (13)	0.0036 (13)	0.0051 (12)
C21	0.0196 (16)	0.0198 (15)	0.0180 (17)	−0.0020 (13)	−0.0003 (13)	0.0016 (13)
Na1	0.0209 (6)	0.0316 (7)	0.0194 (7)	0.0071 (5)	0.0034 (5)	0.0104 (5)
S2	0.0263 (5)	0.0274 (4)	0.0254 (5)	−0.0001 (3)	−0.0056 (4)	0.0073 (4)
O1S	0.0366 (16)	0.098 (3)	0.0368 (17)	−0.0043 (16)	−0.0163 (13)	0.0204 (17)
C1S	0.030 (2)	0.038 (2)	0.039 (2)	−0.0021 (16)	0.0134 (17)	0.0039 (17)
C2S	0.033 (2)	0.046 (2)	0.029 (2)	0.0064 (17)	0.0096 (17)	0.0072 (17)
O1W	0.0214 (12)	0.0267 (13)	0.0211 (13)	0.0055 (11)	0.0001 (10)	0.0051 (10)

### Geometric parameters (Å, º)

**Table:**

Cl1—C4	1.744 (3)	C12—C13	1.463 (5)
S1—O1	1.445 (2)	C13—C14	1.358 (4)
S1—O2	1.449 (2)	C13—C21	1.487 (4)
S1—O3	1.461 (2)	C14—C15	1.437 (4)
S1—C2	1.792 (3)	C14—H14	0.9500
N1—N2	1.321 (4)	C15—C16	1.407 (4)
N1—C1	1.395 (4)	C15—C20	1.408 (5)
N1—H1	0.82 (4)	C16—C17	1.369 (5)
N2—C11	1.324 (4)	C16—H16	0.9500
O2—Na1	2.614 (3)	C17—C18	1.396 (5)
O3—Na1^i^	2.353 (2)	C17—H17	0.9500
O4—C12	1.244 (4)	C18—C19	1.384 (5)
O4—Na1	2.283 (2)	C18—H18	0.9500
O5—C21	1.215 (4)	C19—C20	1.402 (4)
O5—Na1	2.548 (3)	C19—H19	0.9500
O6—C21	1.320 (4)	Na1—O1W^ii^	2.408 (3)
O6—H6	0.92 (5)	Na1—O1W	2.432 (3)
C1—C6	1.395 (4)	Na1—S2^iii^	3.3866 (15)
C1—C2	1.410 (4)	Na1—Na1^ii^	3.782 (2)
C2—C3	1.392 (4)	S2—O1S	1.496 (3)
C3—C4	1.380 (4)	S2—C1S	1.774 (4)
C3—H3	0.9500	S2—C2S	1.778 (4)
C4—C5	1.393 (4)	C1S—H1S1	0.9800
C5—C6	1.390 (4)	C1S—H1S2	0.9800
C5—C7	1.504 (4)	C1S—H1S3	0.9800
C6—H6A	0.9500	C2S—H2S1	0.9800
C7—H7A	0.9800	C2S—H2S2	0.9800
C7—H7B	0.9800	C2S—H2S3	0.9800
C7—H7C	0.9800	O1W—H1WA	0.80 (5)
C11—C20	1.464 (4)	O1W—H1WB	0.84 (6)
C11—C12	1.467 (4)		
			
O1—S1—O2	113.86 (13)	C17—C18—H18	119.7
O1—S1—O3	112.63 (13)	C18—C19—C20	119.9 (3)
O2—S1—O3	112.10 (13)	C18—C19—H19	120.0
O1—S1—C2	105.64 (13)	C20—C19—H19	120.0
O2—S1—C2	107.23 (13)	C19—C20—C15	119.1 (3)
O3—S1—C2	104.56 (13)	C19—C20—C11	122.1 (3)
N2—N1—C1	119.8 (3)	C15—C20—C11	118.8 (3)
N2—N1—H1	115 (3)	O5—C21—O6	122.3 (3)
C1—N1—H1	125 (3)	O5—C21—C13	124.8 (3)
N1—N2—C11	120.3 (3)	O6—C21—C13	112.9 (3)
S1—O2—Na1	141.39 (13)	O4—Na1—O3^iv^	111.35 (10)
S1—O3—Na1^i^	140.63 (13)	O4—Na1—O1W^ii^	149.04 (11)
C12—O4—Na1	147.7 (2)	O3^iv^—Na1—O1W^ii^	85.77 (8)
C21—O5—Na1	137.0 (2)	O4—Na1—O1W	94.69 (9)
C21—O6—H6	112 (3)	O3^iv^—Na1—O1W	151.14 (10)
N1—C1—C6	120.5 (3)	O1W^ii^—Na1—O1W	77.20 (9)
N1—C1—C2	120.0 (3)	O4—Na1—O5	67.73 (8)
C6—C1—C2	119.5 (3)	O3^iv^—Na1—O5	83.28 (8)
C3—C2—C1	118.4 (3)	O1W^ii^—Na1—O5	90.04 (9)
C3—C2—S1	116.2 (2)	O1W—Na1—O5	119.38 (10)
C1—C2—S1	125.3 (2)	O4—Na1—O2	68.36 (8)
C4—C3—C2	120.8 (3)	O3^iv^—Na1—O2	98.90 (8)
C4—C3—H3	119.6	O1W^ii^—Na1—O2	136.49 (9)
C2—C3—H3	119.6	O1W—Na1—O2	78.79 (9)
C3—C4—C5	121.9 (3)	O5—Na1—O2	133.44 (8)
C3—C4—Cl1	118.0 (2)	O4—Na1—S2^iii^	137.82 (8)
C5—C4—Cl1	120.1 (2)	O3^iv^—Na1—S2^iii^	71.38 (6)
C6—C5—C4	117.2 (3)	O1W^ii^—Na1—S2^iii^	71.14 (7)
C6—C5—C7	121.1 (3)	O1W—Na1—S2^iii^	81.06 (7)
C4—C5—C7	121.8 (3)	O5—Na1—S2^iii^	149.09 (7)
C5—C6—C1	122.2 (3)	O2—Na1—S2^iii^	69.67 (6)
C5—C6—H6A	118.9	O4—Na1—Na1^ii^	126.76 (9)
C1—C6—H6A	118.9	O3^iv^—Na1—Na1^ii^	121.08 (8)
C5—C7—H7A	109.5	O1W^ii^—Na1—Na1^ii^	38.83 (6)
C5—C7—H7B	109.5	O1W—Na1—Na1^ii^	38.37 (6)
H7A—C7—H7B	109.5	O5—Na1—Na1^ii^	108.42 (8)
C5—C7—H7C	109.5	O2—Na1—Na1^ii^	109.67 (7)
H7A—C7—H7C	109.5	S2^iii^—Na1—Na1^ii^	72.20 (4)
H7B—C7—H7C	109.5	O1S—S2—C1S	105.97 (19)
N2—C11—C20	116.4 (3)	O1S—S2—C2S	106.36 (19)
N2—C11—C12	123.4 (3)	C1S—S2—C2S	97.93 (17)
C20—C11—C12	120.2 (3)	O1S—S2—Na1^iii^	103.09 (14)
O4—C12—C13	122.4 (3)	C1S—S2—Na1^iii^	109.51 (13)
O4—C12—C11	119.5 (3)	C2S—S2—Na1^iii^	131.85 (13)
C13—C12—C11	118.1 (3)	S2—C1S—H1S1	109.5
C14—C13—C12	119.5 (3)	S2—C1S—H1S2	109.5
C14—C13—C21	120.7 (3)	H1S1—C1S—H1S2	109.5
C12—C13—C21	119.8 (3)	S2—C1S—H1S3	109.5
C13—C14—C15	123.9 (3)	H1S1—C1S—H1S3	109.5
C13—C14—H14	118.1	H1S2—C1S—H1S3	109.5
C15—C14—H14	118.1	S2—C2S—H2S1	109.5
C16—C15—C20	119.9 (3)	S2—C2S—H2S2	109.5
C16—C15—C14	120.5 (3)	H2S1—C2S—H2S2	109.5
C20—C15—C14	119.6 (3)	S2—C2S—H2S3	109.5
C17—C16—C15	120.1 (3)	H2S1—C2S—H2S3	109.5
C17—C16—H16	119.9	H2S2—C2S—H2S3	109.5
C15—C16—H16	119.9	Na1^ii^—O1W—Na1	102.80 (9)
C16—C17—C18	120.3 (3)	Na1^ii^—O1W—H1WA	111 (3)
C16—C17—H17	119.9	Na1—O1W—H1WA	111 (3)
C18—C17—H17	119.9	Na1^ii^—O1W—H1WB	128 (4)
C19—C18—C17	120.7 (3)	Na1—O1W—H1WB	100 (3)
C19—C18—H18	119.7	H1WA—O1W—H1WB	103 (5)
			
C1—N1—N2—C11	177.3 (3)	Na1—O4—C12—C11	−172.1 (3)
O1—S1—O2—Na1	109.0 (2)	N2—C11—C12—O4	1.6 (5)
O3—S1—O2—Na1	−20.3 (2)	C20—C11—C12—O4	−179.9 (3)
C2—S1—O2—Na1	−134.49 (19)	N2—C11—C12—C13	−178.0 (3)
O1—S1—O3—Na1^i^	118.6 (2)	C20—C11—C12—C13	0.4 (4)
O2—S1—O3—Na1^i^	−111.4 (2)	O4—C12—C13—C14	179.9 (3)
C2—S1—O3—Na1^i^	4.4 (2)	C11—C12—C13—C14	−0.4 (4)
N2—N1—C1—C6	6.2 (4)	O4—C12—C13—C21	−0.5 (5)
N2—N1—C1—C2	−173.9 (3)	C11—C12—C13—C21	179.2 (3)
N1—C1—C2—C3	178.7 (3)	C12—C13—C14—C15	0.7 (5)
C6—C1—C2—C3	−1.5 (4)	C21—C13—C14—C15	−178.9 (3)
N1—C1—C2—S1	−3.8 (4)	C13—C14—C15—C16	178.2 (3)
C6—C1—C2—S1	176.0 (2)	C13—C14—C15—C20	−1.0 (5)
O1—S1—C2—C3	−37.7 (3)	C20—C15—C16—C17	−0.5 (5)
O2—S1—C2—C3	−159.4 (2)	C14—C15—C16—C17	−179.7 (3)
O3—S1—C2—C3	81.4 (2)	C15—C16—C17—C18	0.4 (5)
O1—S1—C2—C1	144.8 (3)	C16—C17—C18—C19	−0.2 (5)
O2—S1—C2—C1	23.0 (3)	C17—C18—C19—C20	0.2 (5)
O3—S1—C2—C1	−96.2 (3)	C18—C19—C20—C15	−0.3 (5)
C1—C2—C3—C4	0.7 (4)	C18—C19—C20—C11	178.3 (3)
S1—C2—C3—C4	−177.0 (2)	C16—C15—C20—C19	0.5 (4)
C2—C3—C4—C5	0.9 (5)	C14—C15—C20—C19	179.7 (3)
C2—C3—C4—Cl1	−179.8 (2)	C16—C15—C20—C11	−178.2 (3)
C3—C4—C5—C6	−1.6 (4)	C14—C15—C20—C11	1.0 (4)
Cl1—C4—C5—C6	179.1 (2)	N2—C11—C20—C19	−0.8 (4)
C3—C4—C5—C7	178.3 (3)	C12—C11—C20—C19	−179.4 (3)
Cl1—C4—C5—C7	−1.0 (4)	N2—C11—C20—C15	177.8 (3)
C4—C5—C6—C1	0.8 (4)	C12—C11—C20—C15	−0.7 (4)
C7—C5—C6—C1	−179.1 (3)	Na1—O5—C21—O6	173.8 (2)
N1—C1—C6—C5	−179.4 (3)	Na1—O5—C21—C13	−5.9 (5)
C2—C1—C6—C5	0.8 (4)	C14—C13—C21—O5	−179.7 (3)
N1—N2—C11—C20	−176.5 (3)	C12—C13—C21—O5	0.8 (5)
N1—N2—C11—C12	2.0 (4)	C14—C13—C21—O6	0.6 (4)
Na1—O4—C12—C13	7.6 (6)	C12—C13—C21—O6	−178.9 (3)

Symmetry codes: (i) *x*+1, *y*, *z*; (ii) −*x*, −*y*+1, −*z*; (iii) −*x*−1, −*y*+1, −*z*; (iv) *x*−1, *y*, *z*.

### Hydrogen-bond geometry (Å, º)

**Table:**

*D*—H···*A*	*D*—H	H···*A*	*D*···*A*	*D*—H···*A*
N1—H1···O2	0.82 (4)	2.14 (4)	2.747 (3)	131 (3)
N1—H1···O4	0.82 (4)	1.84 (4)	2.532 (4)	141 (4)
O6—H6···O1*S*	0.92 (5)	1.67 (5)	2.575 (4)	168 (4)
O1*W*—H1*WA*···O5^i^	0.80 (5)	2.33 (5)	2.944 (3)	134 (4)
O1*W*—H1*WA*···O1*S*^i^	0.80 (5)	2.48 (5)	3.138 (5)	140 (4)
O1*W*—H1*WB*···O3	0.84 (6)	2.11 (6)	2.942 (3)	176 (5)

Symmetry code: (i) *x*+1, *y*, *z*.
